# Effects of working memory load on frontal connectivity in children with autism spectrum disorder: a fNIRS study

**DOI:** 10.1038/s41598-022-05432-3

**Published:** 2022-01-27

**Authors:** Yvonne M. Y. Han, Ming-Chung Chan, Melody M. Y. Chan, Michael K. Yeung, Agnes S. Chan

**Affiliations:** 1 Department of Rehabilitation Sciences, The Hong Kong Polytechnic University, Hung Hom, Kowloon, Hong Kong SAR People’s Republic of China; 2 University Research Facility in Behavioral and Systems Neuroscience (UBSN), The Hong Kong Polytechnic University, Kowloon, Hong Kong SAR People’s Republic of China; 3 Department of Psychology, The Chinese University of Hong Kong, Kowloon, Hong Kong SAR People’s Republic of China

**Keywords:** Autism spectrum disorders, Brain

## Abstract

Individuals with autism spectrum disorder (ASD) perform poorly in working memory (WM) tasks, with some literature suggesting that their impaired performance is modulated by WM load. While some neuroimaging and neurophysiological studies have reported altered functional connectivity during WM processing in individuals with autism, it remains largely unclear whether such alterations are moderated by WM load. The present study aimed to examine the effect of WM load on functional connectivity within the prefrontal cortex (PFC) in ASD using functional near-infrared spectroscopy (fNIRS). Twenty-two children with high-functioning ASD aged 8–12 years and 24 age-, intelligent quotient (IQ)-, sex- and handedness-matched typically developing (TD) children performed a number *n*-back task with three WM loads (0-back, 1-back, and 2-back). Hemodynamic changes in the bilateral lateral and medial PFC during task performance were monitored using a multichannel NIRS device. Children with ASD demonstrated slower reaction times, specifically during the “low load” condition, than TD children. In addition, the ASD and TD groups exhibited differential load-dependent functional connectivity changes in the lateral and medial PFC of the right but not the left hemisphere. These findings indicate that WM impairment in high-functioning ASD is paralleled by load-dependent alterations in right, but not left, intrahemispheric connectivity during WM processing in children with ASD. A disruption of functional neural connections that support different cognitive processes may underlie poor performance in WM tasks in ASD.

## Introduction

Autism spectrum disorder (ASD) is a neurodevelopmental disorder characterized by disturbances in social communication, as well as manifestations of restricted and stereotyped behavior^1^. It has been suggested that deficiencies in working memory (WM), which is the temporary storage system that serves as a basis for complex cognitive functions^2^, and its associated executive functioning deficits^3^, contribute to ASD symptom manifestation and daily dysfunction^4–7^. Specifically, individuals with ASD are found to exhibit specific face recognition impairment when they are asked to recall the faces presented a few trials before^8,9^ but not face matching tasks^10^. Moreover, when children with ASD learn, they are presented with difficulties integrating and generalizing learned knowledge, especially when the complexity of knowledge increases^11^. Many individual studies have demonstrated deficient WM in individuals with ASD, but the findings have been inconsistent: some studies have found a significant difference in WM task performance between individuals with ASD and typically developing (TD) controls^12–15^, whereas others have not^16–18^. Nevertheless, a recent meta-analysis confirmed the presence of WM deficits in ASD^19^, with previous researchers suggesting that the level of WM impairment might be associated with the increase in the complexity of information and the demand for WM capacity involved in a task^19,20^.

It is well established that the frontal lobe is involved in WM. Evidence for this comes from studies of the *n*-back task, one of the most widely adopted paradigms reflecting WM ability in cognitive neuroscience. For instance, an aggregate of studies has shown bilateral activation in the dorsolateral and ventrolateral PFC, dorsal cingulate cortex, medial premotor cortex, and parietal cortex during the n-back task^21^. Additionally, functional coupling between frontal and parietal regions^22^ and between the left and right PFC^23^ increases during processing, which requires increased WM demand. These studies provide evidence to support the notion that WM is mediated by the PFC and its distributed neural networks^24–26^, which also implies that good performance in a WM task requires an efficient flow of information between these brain areas to maintain and integrate information.

Frontal lobe dysfunction in ASD has long been documented in the literature. For example, a neuroimaging meta-analysis showed that children with high-functioning ASD showed hypoactivation in the right inferior frontal gyrus and right anterior cingulate gyrus during cognitive tasks involving inhibition, switching and updating components^27^. In addition, Narita, Saotome^28^ reported abnormal activation patterns in ASD adults over the left dorsolateral prefrontal cortex (PFC) during a visuospatial delayed recall task, a task reflecting participants’ WM capacity. These studies collectively imply that abnormal frontal activity might be associated with impaired cognitive performance in ASD.

Furthermore, converging evidence suggests altered functional connectivity of the brain involving the PFC in both resting and task states in ASD, although it is still controversial whether these alterations should be best characterized as global underconnectivity and/or local overconnectivity^29–31^. Since functional coupling between and within regions in the brain, especially the PFC, is important for the execution of WM tasks, altered connectivity of the brain may underlie the WM deficits exhibited by individuals with ASD. Indeed, there have been some studies reporting altered functional connectivity during *n*-back tasks in ASD. Some early fMRI studies found that adults with ASD exhibited overall underconnectivity between the PFC and parietal regions during a letter 2-back task^32^ and between the frontal and fusiform or parietal areas across different WM load levels in a facial *n*-back task^33^. Additionally, a more recent magnetoencephalography study found reduced synchronization within frontotemporal networks during a visuospatial 2-back task in children with ASD compared to TD children^34^. Despite these findings, it remains largely unclear whether the altered functional connectivity during WM task performance in individuals with ASD is moderated by WM load. Thus, the aim of the present study was to examine the effect of WM load on functional connectivity in ASD.

Functional near-infrared spectroscopy (fNIRS) is a noninvasive neuroimaging technique that monitors changes in the concentrations of oxyhemoglobin (HbO) and deoxyhemoglobin (HbR), which are associated with activities of the cerebral cortex^35^. During fNIRS recording, a fNIRS device emits at least two near-infrared lights with a wavelength of 650–900 nm into the scalp. The lights then penetrate the scalp, skull, cerebrospinal fluid, and brain tissue in a banana-shaped trajectory, and the exiting lights are recorded by a receiver. Variations in optical density caused by changes in the brain’s metabolism can then be used to estimate the changes in hemoglobin concentration in the sampled brain issue via application of the modified Beer-Lambert law^36^. Over the last decade, fNIRS has been used to assess the functional coupling of brain regions, especially the PFC, during the performance of a wide variety of tasks. For example, fNIRS has been used to measure functional connectivity between two cortical areas that underlie motor performance^37^. It has also been used to measure intrahemispheric and/or interhemispheric connectivity within the dorsolateral PFC and frontal pole during the *n*-back task in healthy young adults^38^.

It is widely agreed that fNIRS possesses adequate temporal and spatial resolution and relatively high tolerance to motion and environmental conditions^39^. These unique properties make fNIRS a suitable tool for studying functional connectivity in pediatric populations. In the present study, we utilized fNIRS to examine functional connectivity within the PFC during the *n*-back task with three WM loads in children with high-functioning ASD. We hypothesized that compared to TD controls, children with ASD would exhibit WM impairment and altered PFC connectivity that is load-dependent during *n*-back task performance.

## Method

### Participants

This study was conducted in accordance with the Declaration of Helsinki. The research protocol was approved by the Human Subjects Ethics Sub-Committee of Hong Kong Polytechnic University. Twenty-two children with ASD and 24 TD children aged 8–12 years voluntarily participated in this study. These children were recruited through advertisements sent to parent groups via social media (e.g., WhatsApp and Facebook) and posted in the rehabilitation clinic at Hong Kong Polytechnic University, as well as through invitation letters sent to teachers of local primary schools. All potential participants were screened for eligibility prior to the study, and those with a history of epilepsy or head trauma were excluded. A participant was included in the high-functioning ASD group if he or she fulfilled the following criteria: (1) he or she had received a diagnosis of ASD based on the *Diagnostic and Statistical Manual of Mental Disorders–5th Edition* (DSM-5;^1^) from psychiatrists before the commencement of this study and scored above the cutoff points in all subscales in the Autism Diagnostic Interview-Revised (ADI-R;^40^) that was conducted by a registered clinical psychologist during the screening session for this study and (2) obtained a full-scale intelligent quotient (IQ) ≥ 80 on the Hong Kong version of the Wechsler Intelligence Scale for Children-Fourth Edition short form [WISC-IV (HK);^41^], which was also administered by a clinical psychologist during the screening session. For a participant to be included in the TD group, he or she should have no history of developmental delay or any other neurological/psychiatric disorders.

### Procedure and materials

All children and their parents attended individual screening and neuropsychological/neurophysiological measurement sessions at The Hong Kong Polytechnic University. Prior to the screening and assessment, the children and their parents were informed about the assessment procedures, and informed consent was obtained from the children and parents. All of the child participants were administered the Tumbling “E” Test to ensure that they had normal or corrected-to-normal vision before any cognitive assessment and fNIRS testing. The child participants were screened for intellectual functioning by a clinical psychologist, followed by fNIRS recording sessions conducted by trained research assistants. These procedures were conducted in separate rooms. Short breaks were given to the participants every 30 min, and the entire experiment lasted for approximately two hours for each participant.

The demographic information of the participants was collected during interviews conducted by a clinical psychologist with parents in a separate room. Notably, we collected the medication history of the participants to delineate any possible mediating effects of some psychiatric medications on EF functioning, especially for working memory (e.g., medication for alleviating attention-deficit/hyperactivity symptoms in ASD^42^), during our data analysis. To measure participants’ level of autistic traits in both the ASD and TD groups, the Social Responsiveness Scale–Second Edition (SRS-2) was administered^43^. The SRS-2 is a sensitive measure of children’s social impairment related to ASD over the past six months. It consists of five subscales that measure social awareness, social cognition, social communication, social motivation, restricted interests and repetitive behavior. Each item is rated on a four-point Likert scale from 1 (not true) to 4 (almost always true). Higher total scores indicate greater difficulties in socialization. For the assessment of IQ, the WISC-IV (HK) short form was adopted in this study, which comprises two verbal subtests, digit span and similarities, and two performance subtests, matrix reasoning and coding, yielding a full IQ score with a mean of 100 and a standard deviation of 15.

### *N*-back paradigm

**Figure 1:**
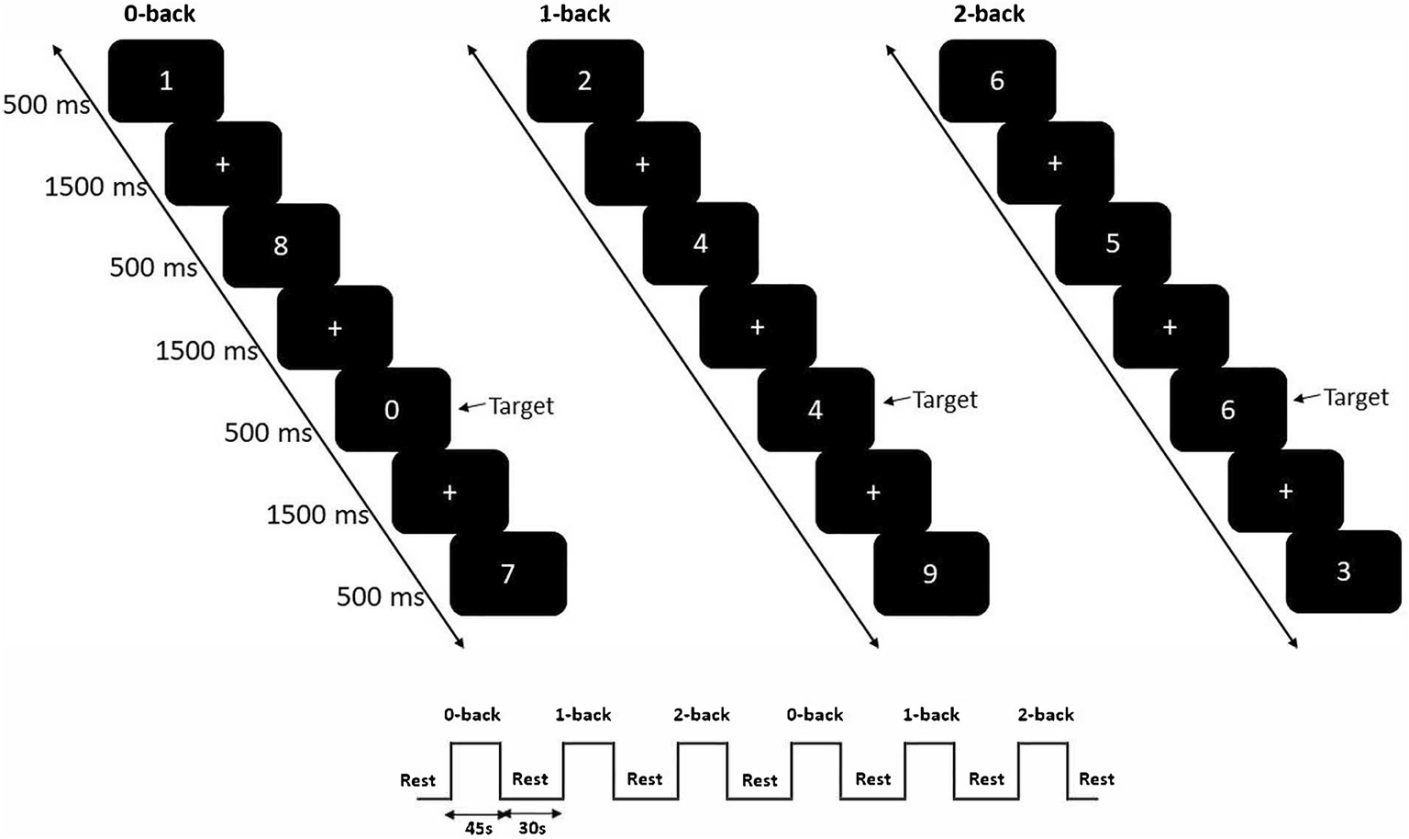
Number n-back paradigm used in this study.

Each participant performed the *n*-back task, which was run by E-prime 2.0 Software (Psychology Software Tools, Pittsburgh, PA, USA), while prefrontal hemodynamic changes were recorded by fNIRS. The *n*-back task was adapted from previous fNIRS studies^44–46^ and consisted of the 0-back (i.e., low WM load), 1-back (i.e., medium WM load), and 2-back (i.e., high WM load) conditions (Fig. 1). Each condition was presented twice, and the three tasks alternated in blocks (i.e., 0-1-2-0-1-2, 0-2-1-0-2-1, 1-0-2-1-0-2, 1-2-0-1-2-0, 2-0-1-2-0-1 or 2-1-0-2-1-0) and were separated by 30-s rest blocks during which the participants sat still with their eyes open. The order was counterbalanced across participants to prevent order effects. Each task block consisted of a 5-s instruction cue that introduced the task, followed by 20 trials (5 target and 15 nontarget trials) presented in a pseudorandom manner.

In the 0-back condition, the participants were instructed to press the left button of a mouse when the digit “0” (i.e., target) appeared but to press the right button for other digits. In the 1-back condition, the participants were instructed to press the left button when the presented digit was identical to the digit presented in the previous trial (i.e., target) or otherwise, the right button. In the 2-back condition, the participants were instructed to press the left button when the presented digit was identical to the digit presented two trials before (i.e., target) or otherwise, the right button. Each digit was presented for 500 ms, followed by an interstimulus interval of 1500 ms. Each task block lasted for 45 s, and the total duration was 8 min.

### fNIRS measurement

The fNIRS recording session was conducted in a quiet, dim-lighted room^47^. Each participant sat 60 cm away from a 15’’ LCD monitor that was used for stimulus display. Their head dimensions (nasion–inion and left–right periauricular points) were measured to facilitate offline spatial registration of fNIRS channels^48^, where the channel positions were transformed into Montreal Neurological Institute (MNI) space and projected onto the surface of a volume rendered children brain template chosen according to participants’ age and head dimensions^49^.

Prefrontal hemodynamics during the *n*-back task were measured by a 52-channel fNIRS optical topography system (ETG-4000; Hitachi Medical Co., Tokyo, Japan). It consisted of 17 sources of two wavelengths (695 and 830 nm) and 16 detectors. A custom-built headband mounted with optical emitters and receivers, which were arranged in a 3 × 11 matrix, was placed on the children’s heads based on the 10–10 system^50^, an extension to the international 10–20 system^51^. The probe in the center of the lowest row was placed at Fpz, guided by a reference point marked on the headband that was standardized to be placed over the nasion of all subjects. The sampling frequency was 10 Hz. Each pair of sources and detectors was 3 cm apart, and therefore, brain activity was measured approximately 15–20 mm beneath the scalp^52^.

### Statistical analysis

#### Demographic and clinical data

Demographic data including age, IQ, sex and handedness, as well as clinical data including SRS-2 total score of the two groups, were compared using independent-sample t-tests after normality of data was checked. Descriptive data of medication history and ADI-R domain scores of ASD participants were also reported. To delineate the possible effects of medications on n-back task performance, we planned to perform additional subgroup analyses for both behavioral and fNIRS data by excluding participants who are currently on a drug regimen regardless of ADHD/antipsychotic medications.

#### Behavioral data

To comprehensively examine WM functioning in ASD, apart from n-back behavioral data, we also compared the WISC-IV (HK) subscores of forward and backward digit spans between ASD and TD groups using independent-sample t-tests. For WM data, the mean latency time for all correct trials for each subject was calculated and named the mean reaction time (RT), and the percentage of correct trials as accuracy (ACC) for the *n*-back task was analyzed. We first inspected the normality of the RT and ACC distributions with Shapiro–Wilk tests. Because RT variables in all three conditions were positively skewed, they were log-transformed. Given that most log-transformed RT variables no longer violated the normal distribution, *p*s > 0.05, parametric tests were used for analysis. For each *n*-back condition, ACC and mean RT were separately analyzed with two-way mixed-design analysis of variance (ANOVA) with load (low, medium, and high) as the within-subjects factor and group (ASD and TD) as the between-subjects factor.

#### fNIRS processing

**Figure 2:**
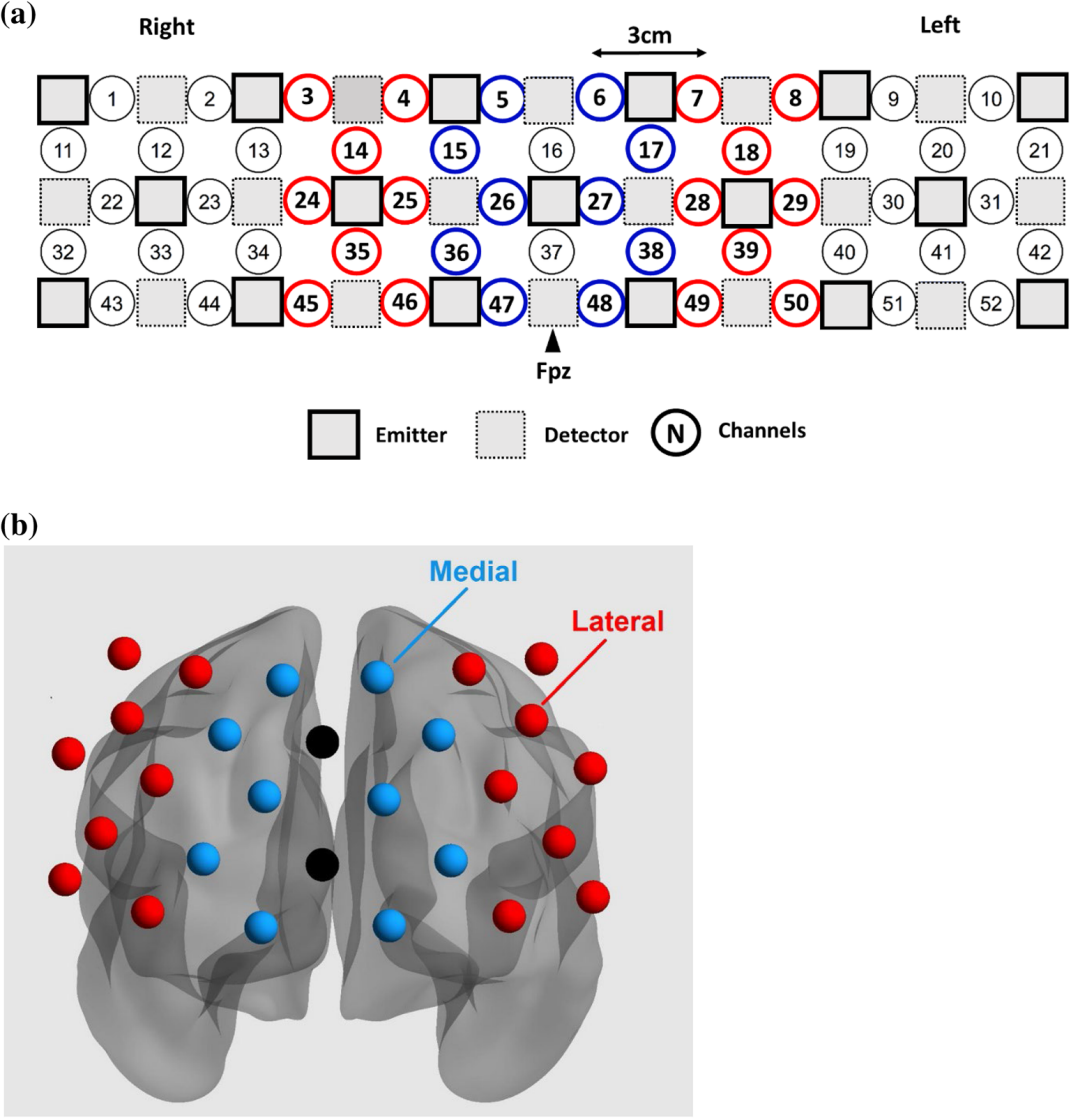
Arrangement of channels (**a**) in the sensor space and (**b**) in the brain space. Channels in red and blue represent the lateral and medial prefrontal cortex, respectively.

We performed functional connectivity followed by activation analyses. For both analyses, the raw fNIRS signals were first converted to optical density changes and then transformed into changes in HbO and HbR using the modified Beer-Lambert law^36^. Since HbO has a higher signal-to-noise ratio than HbR^52–54^, only the HbO data were analyzed. While we focused on the lateral PFC because this region has been implicated in *n*-back task performance^21^, the medial frontopolar region was also examined to assess the specificity of the results. Based on the virtual registration of channels^48^, eight and five channels represented the lateral PFC and medial frontopolar cortex on each side, respectively (Fig. 2). Channels 7, 8, 18, 28, 29, 39, 49, and 50 represented the left lateral PFC; channels 3, 4, 14, 24, 25, 35, 45, and 46 represented the right lateral PFC; channels 6, 17, 27, 38, and 48 represented the left medial PFC; and channels 5, 15, 26, 36, and 47 represented the right medial PFC. The outermost channels covering the temporal lobe regions were not analyzed because of poor optode–scalp contact for most of the children.

#### Functional connectivity analysis

The connectivity patterns during the *n*-back task were analyzed using the NIRS Brain AnalyzIR Toolbox^55^ for MATLAB (The Mathworks, Natick, MA). The traditional approach to connectivity analysis computes the Pearson correlations between possible pairs of channels, but this approach did not address systemic physiological noise and head motion, which are temporally correlated (colored) noise in the fNIRS signals and may affect the accuracy of the results^56^. In this case, a prewhitening filter can be used to remove the autocorrelation and whiten the frequency content of the signal^57^. First, temporally correlated physiological artifacts were corrected by applying an autoregressive prewhitening filter to the signal (*Y*) received by each channel, defined as $Y_{{\left\{ t \right\}}} = \mathop \sum \limits_{i = 1}^{P} a_{i} \cdot Y_{{\left\{ {t - i} \right\}}} + \varepsilon_{{\left\{ t \right\}}}$ and $\varepsilon_{{\left\{ t \right\}}} \in N\left( {0,\sigma^{2} } \right)$, where t indicates the sample point, the set $a_{i }$ is the autoregressive coefficient of the model, and P is the model order that was selected based on Bayesian information criteria (BIC). This equation states that the current sample point (Y{t}) can be predicted based on the last several time points (a1.Y{t − 1}… ap.Y{t − p}) and newly added information at that time point, which are the innovations and denoted as (ε{t}). The innovations can be thought of as new information that is added to the total signal at each time point. This method yielded prewhitened signals without autocorrelations with time^57,58^. The prewhitened signals were then processed using the procedure described in Santosa et al.^56^ to remove motion artifacts. Then, correlation coefficients (*R*) between two prewhitened and preweighted signals (denoted as *A*_*S,W*_ and *B*_*S,W*_ below) were calculated using the equation $R = b_{1} \cdot \frac{{\sigma_{A} }}{{\sigma_{B} }}$ (where $\sigma_{A}$ and $\sigma_{B}$ are the standard deviations of the signals A and B) after obtaining the value $b_{1}$ estimated by applying a least-square solution to the regression model $A_{S,W} = b_{0} + B_{S,W} \cdot b_{1}$. This process was repeated until the correlation coefficients of all possible channel pairs were calculated. The correlation coefficients for each channel pair were Fisher’s *Z*-transformed before averaging across channel pairs.

**Figure 3:**
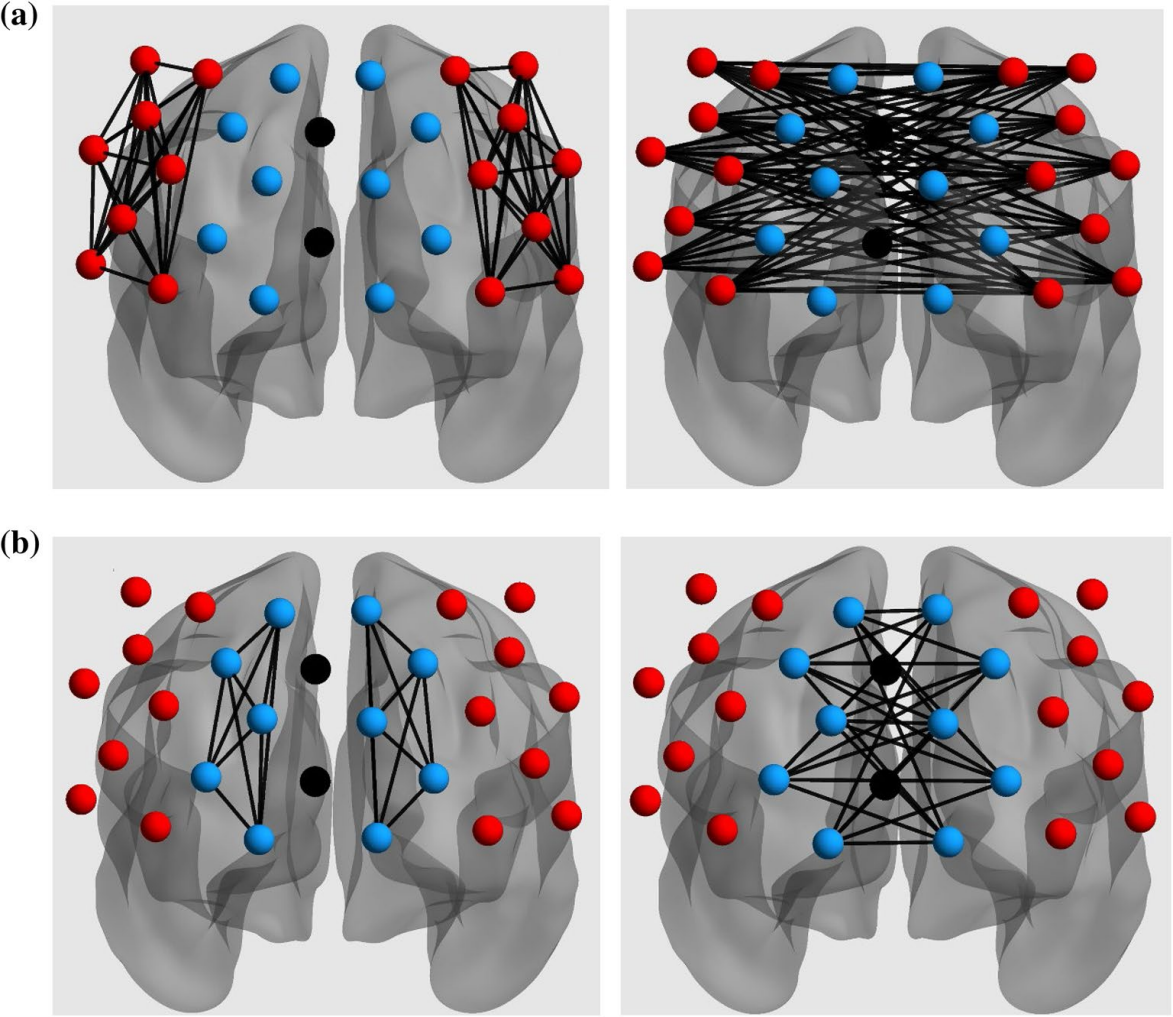
Channel pairs for the intrahemispheric (left) and interhemispheric (right) connectivity measures in the (**a**) lateral (red) and (**b**) medial (blue) prefrontal cortex.

Intrahemispheric and interhemispheric connectivity were examined separately for the lateral and medial PFC. The channel pairs for each connectivity measure are presented in Fig. 3. First, a mixed ANOVA with load (low, medium, and high), region (medial and lateral) and hemisphere (left and right) as within-subjects factors and group (ASD and TD) as a between-subjects factor was conducted on the mean *Z*-transformed correlation coefficients for intrahemispheric connectivity. Then, another mixed ANOVA with load and region as within-subjects factors and group as a between-subjects factor was conducted on the mean *Z*-transformed correlation coefficients for interhemispheric connectivity. Given that previous studies have shown that neural connectivity in ASD exhibits abnormal lateralization patterns that are associated with autism severity^59,60^, we calculated the laterality index with intrahemispheric functional connectivity values. The Z-transformed correlation coefficients for all possible channel pairs of each participant were first summed within each region of interest (ROI), i.e.*.* right medial/lateral PFC and left medial/lateral PFC, with laterality index calculated by the following formula^61^: (L total – R total)/(L total + R total). A higher laterality index indicated more left lateralization during a given task condition. A mixed ANOVA with load (low, medium, and high), region (lateral, medial) and group was performed to explore the differences in laterality between two groups across various WM conditions.

#### Activation analysis

To complement the connectivity analysis, task-related changes in HbO were also examined. The fNIRS signals between each source and detector pair were analyzed using a general linear regression model (GLM) to test for differences between the baseline (30-s rest period at the beginning of the experiment) and the task condition. This can be expressed by the equation Y = X × β + e, where X is the timing of stimulus events, β is the coefficient (weight) of the stimulus condition, and Y is the vector of measurements. Several modules can be used to obtain β, and the AR-IRIS method is the one we used in the paper; this method can remove artifacts and control type-I errors in the fNIRS model (see^55^ for details). These values were then averaged across repetitions for each WM load and across channels for the lateral and medial PFC separately. For each region, a mixed ANOVA with load, hemisphere and region as within-subjects factors and group as a between-subjects factor was conducted on β.

For significant interactions and main effects of group in the behavioral and neurophysiological measures, if any, post hoc independent-/paired-sample *t*-tests with Bonferroni corrections for three WM-load conditions (i.e., *p* < .017 for each condition) were conducted.

#### Correlation analysis

To explore the association between the activation/functional connectivity pattern during the WM task and ASD symptomatology, Spearman's rank-order correlations were performed between SRS-2 and significant fNIRS activation/functional connectivity results. As this analysis is exploratory in nature, no statistical adjustments were applied.

## Results

### Sample characteristics

Table 1 shows the demographic, intellectual, and clinical characteristics of the participants. Independent-sample *t*-tests and chi-square tests showed that the TD and ASD groups did not significantly differ in age (*p* = .93), IQ (*p* = .22), sex (*p* = .20) or handedness (*p* = .086). Regarding autism symptomatology indicated by the SRS-2, the children with ASD had a significantly higher SRS-2 total score than the TD individuals (*p* < .001). All ASD children fulfilled the ASD diagnosis with above-cutoff scores in all ADI-R subdomains. Eight ASD participants were undergoing psychiatric medication treatment.

**Table 1:** Demographic, intellectual, and clinical characteristics of TD (n = 24) and ASD (n = 22) individuals.

	Group		*t/χ*^2^	*p*
	TD	ASD		
	*M* (*SD*)	*M* (*SD*)		
Age (years)	10.2 (0.8)	10.1 (1.0)	0.09	.927
Intelligent quotient (IQ)	107.9 (9.2)	102.7 (17.0)	1.26	.218
Sex (male:female)^a^	17:7	19:3	1.63	.202
Handedness (R:L)	21:3	22:0	2.94	.086
SRS-2 total score	43.2 (16.6)	81.2 (18.8)	7.56	< .001***
Medication (Y:N)	–	8^†^:14	–	–
ADI-R Social Interaction (N = 19)	–	13.1 (8.3)	–	–
ADI-R Communication (N = 19)	–	10.6 (5.5)	–	–
ADI-R Restricted, Repetitive Behavior (N = 19)	–	4.7 (2.8)	–	–

ADI-R, Autism Diagnostic Interview-Revised; SRS-2, Social Responsiveness Scale–Second Edition.

^a^Group comparison was conducted by the likelihood ratio test.

***p < .001.

^†^Medication for alleviating attention-deficit/hyperactivity n = 7; antipsychotics for alleviating inflexibility n = 1.

### Behavioral performance

Table 2 presents the WISC-IV (HK) forward and backward digit span data, n-back ACC and mean (log-transformed) RT in each group. Both forward and backward digit span data were found to be nonsignificantly different between the two groups (ps > .40). For the ACC measure, a mixed 3 (load) × 2 (group) ANOVA showed a significant main effect of load (*F*_2,88_ = 69.71, *p* < .001, $\eta_{p}^{2} =$0.61). The main effect of group was not significant (*F*_1,44_ = 1.87, *p* = .18, $\eta_{p}^{2} =$0.04), and no significant load x group interaction effect was observed. Analysis with all TD (n = 24) and drug-free ASD (n = 14) individuals also yielded nonsignificant results (ACC main effect of group: F_1,36_ = 0.45, *p* = .51; ACC load x group interaction: F_2,72_ = 0.76, *p* = .47).

**Table 2:** Working memory performance of TD (n = 24) and ASD (n = 22) individuals in our sample.

	Group		*t*	*p*	*d*
	TD	ASD			
	*M* (*SD*)	*M* (*SD*)			
**WM capacity**					
WISC-IV (HK) Forward digit span	12.8 (2.3)	13.0 (3.1)	0.208	0.836	0.07
WISC-IV (HK) Backward digit span	9.2 (3.1)	8.4 (3.4)	0.845	0.403	0.25
**0-back**					
Accuracy (%)	93.6 (5.1)	93.6 (5.6)	0.12	0.99	0.00
Raw RT (ms)	483.6 (121.1)	580.7 (150.7)	–	–	–
Log-transformed RT (a.u.)	2.67 (0.09)	2.75 (0.10)	2.73	0.009**	0.84
**1-back**					
Accuracy (%)	90.1 (7.9)	86.0 (13.4)	1.27	0.21	0.37
Raw RT (ms)	562.0 (142.6)	677.7 (202.5)	–	–	–
Log-transformed RT (a.u.)	2.74 (0.11)	2.81 (0.13)	2.24	0.030*	0.58
**2-back**					
Accuracy (%)	80.6 (8.3)	75.9 (10.7)	1.67	0.10	0.49
Raw RT (ms)	592.5 (186.8)	741.9 (253.1)	–	–	–
Log-transformed RT (a.u.)	2.75 (0.12)	2.85 (0.15)	2.32	0.025*	0.74

WISC-IV(HK), Hong Kong version of the Wechsler Intelligence Scale for Children-Fourth Edition short form; WM, working memory.

*p < .05, ** p < .01.

For the mean log-transformed RT measure, a 3 (load) × 2 (group) mixed ANOVA showed significant main effects of load (*F*_2,88_ = 35.84, *p* < .001, $\eta_{p}^{2} =$0.45) and group (*F*_1,44_ = 6.53, *p* = .014, $\eta_{p}^{2} =$0.13), with the ASD group having a slower mean log-transformed RT than the TD group. The interaction between group and load was not significant (*F*_2,88_ = 0.33, *p* = .72, $\eta_{p}^{2} =$0.008). Post hoc *t*-tests with Bonferroni corrections showed that the ASD group had significantly slower mean log-transformed RT than the TD group in the 0-back condition (*p* = .009) and trends toward significance in the 1-back (*p* = .030) and 2-back (*p* = .025) conditions that did not survive Bonferroni corrections. Analysis with all TD (n = 24) and drug-free ASD (n = 14) individuals also yielded similar trends (log-transformed RT main effect of load: F_1,36_ = 36.50, *p* < .001; log-transformed RT main effect of group: F_1,36_ = 1.51, *p* = .23; log-transformed RT load x group interaction: F_2,72_ = 11.68, *p* = .002).

### Effect of n-back load on prefrontal functional connectivity

**Figure 4:**
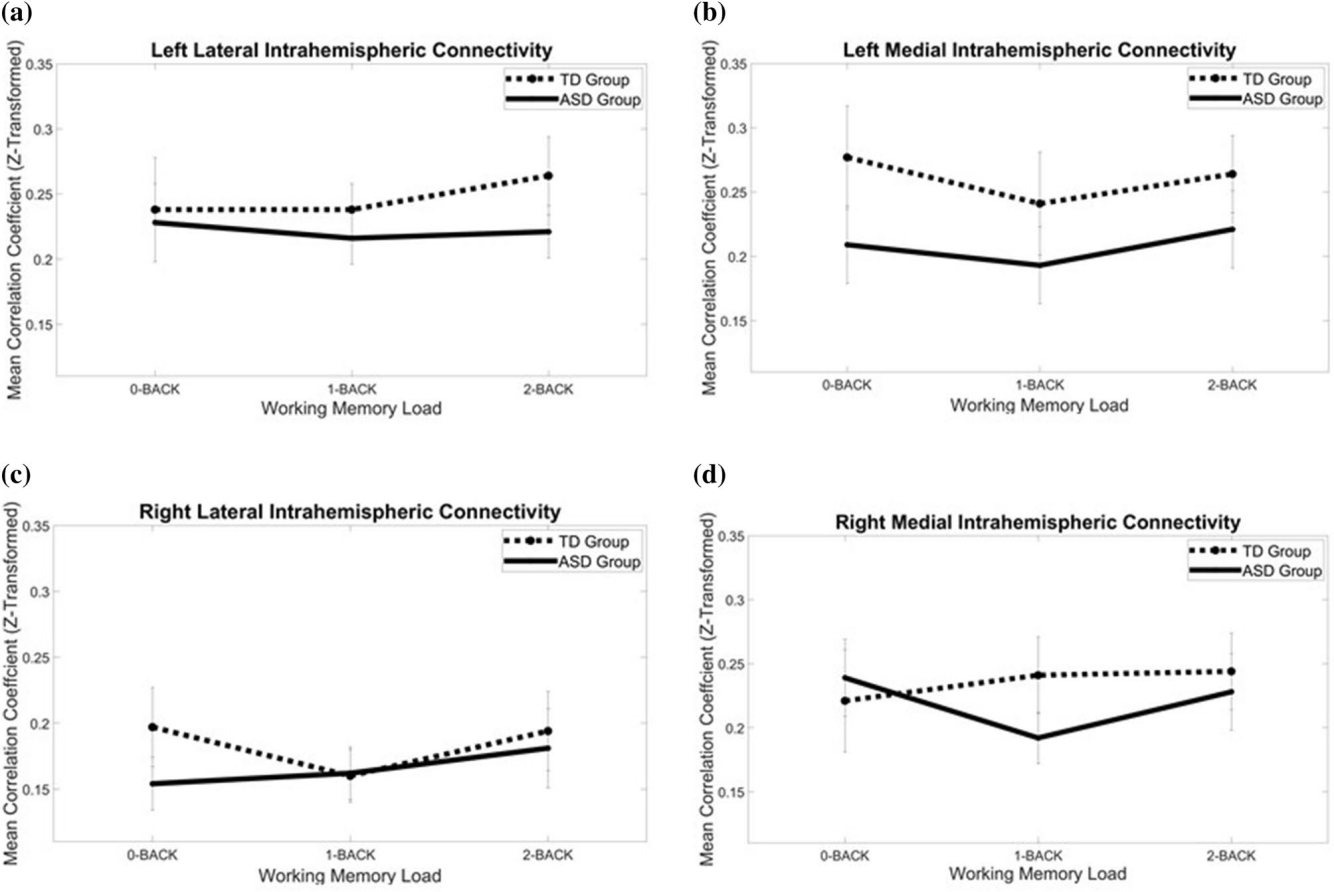
The effect of n-back task load on the (**a**) left lateral, (**b**) left medial, (**c**) right lateral and d) right medial intrahemispheric functional connectivity changes in the prefrontal cortex in TD and ASD.

To clarify the neural mechanisms underlying *n*-back task performance, we analyzed connectivity within and between the left and right medial and lateral PFC (Table 3), which were the regions of interest (ROIs) defined in our study (Fig. 4). Regarding intrahemispheric connectivity, a 2 (hemisphere) × 3 (load) × 2 (region) × 2 (group) mixed ANOVA showed a significant hemisphere × load × region × group effect (*F*_2,88_ = 4.478, *p* = .014, $\eta_{p}^{2} =$0.09). The main effects were significant for hemisphere (*F*_1,44_ = 4.350, *p* = .043, $\eta_{p}^{2} =$0.09) and region (*F*_1,44_ = 4.194, *p* = .047 $\eta_{p}^{2} =$0.09). A follow-up 3 (load) × 2 (region) × 2 (group) mixed ANOVA was conducted for each hemisphere to explore how different loads in the n-back task mediated functional connectivity of the medial and lateral PFC on each side of the brain. In the right hemisphere, a significant load × region × group interaction (*F*_2,88_ = 6.073, *p* = .003, $\eta_{p}^{2} =$0.12) and a significant main effect of region (*F*_1,44_ = 7.351, *p* = .010, $\eta_{p}^{2} =$0.14) were found. Thus, the ASD and TD groups had different connectivity changes in the right hemisphere with load in the lateral and medial PFC. A post hoc paired-sample t-test showed a trend toward significance between 0-back and 1-back connectivity in the right medial regions of the ASD group (*p* = .030), which did not survive Bonferroni correction. On the left side, neither a significant main effect nor a significant interaction was found (ps > .24). Analysis with all TD (n = 24) and drug-free ASD (n = 14) individuals showed similar results (hemisphere x load x region x group 4-way interaction: F_2,72_ = 3.48, *p* = .036; right PFC load x region x group 3-way interaction: F_2,72_ = 4.75, *p* = .012). For the functional connectivity laterality index (Table 4), a 2 (region) × 3 (load) × 2 (group) mixed ANOVA was performed. The main effect was significant for region (F1,44 = 4.118, *p* = .049, ηp^2^ = 0.09), and none of the other main effects, including the main effect of group, were significant. There was also a significant load x region interaction effect (F2,88 = 4.314, *p* = .016, ηp^2^ = 0.09), and none of the other interaction effects with group were significant.

**Table 3:** Connectivity measures indicating (a) intrahemispheric and (b) interhemispheric functional connectivity in the lateral and medial prefrontal cortex during the n-back task in TD (n = 24) and ASD (n = 22) individuals.

	Left hemisphere		Right hemisphere	
	TD	ASD	TD	ASD
	*M* (*SD*)	*M* (*SD*)	*M* (*SD*)	*M* (*SD*)
*(a) Intrahemispheric*				
**Medial region**				
0-back	0.238 (0.177)	0.228 (0.137)	0.197 (0.136)	0.154 (0.083)
1-back	0.238 (0.092)	0.216 (0.090)	0.160 (0.095)	0.162 (0.104)
2-back	0.264 (0.167)	0.221 (0.094)	0.194 (0.138)	0.181 (0.123)
**Lateral region**				
0-back	0.277 (0.173)	0.209 (0.148)	0.221 (0.199)	0.239 (0.143)
1-back	0.241 (0.174)	0.193 (0.161)	0.241 (0.151)	0.192 (0.097)
2-back	0.264 (0.142)	0.221 (0.152)	0.244 (0.148)	0.228 (0.155)

	Medial		Lateral	
	TD	ASD	TD	ASD
	*M* (*SD*)	*M* (*SD*)	*M (SD)*	*M (SD)*
*(b) Interhemispheric*				
0-back	0.270 (0.143)	0.279 (0.119)	0.227 (0.197)	0.190 (0.094)
1-back	0.264 (0.121)	0.243 (0.123)	0.190 (0.093)	0.161 (0.116)
2-back	0.280 (0.132)	0.286 (0.134)	0.214 (0.167)	0.182 (0.124)

**Table 4:** Functional connectivity laterality index of ASD and TD individuals during n-back task.

	Laterality Index			
	Medial		Lateral	
	TD	ASD	TD	ASD
	*M* (*SD*)	*M* (*SD*)	*M (SD)*	*M (SD)*
0-back	0.123 (0.467)	− 0.042 (0.441)	0.041 (0.417)	0.100 (0.392)
1-back	− 0.005 (0.504)	− 0.143 (0.465)	0.169 (0.369)	0.213 (0.418)
2-back	0.060 (0.384)	− 0.108 (0.445)	0.131 (0.446)	0.146 (0.437)

**Figure 5:**
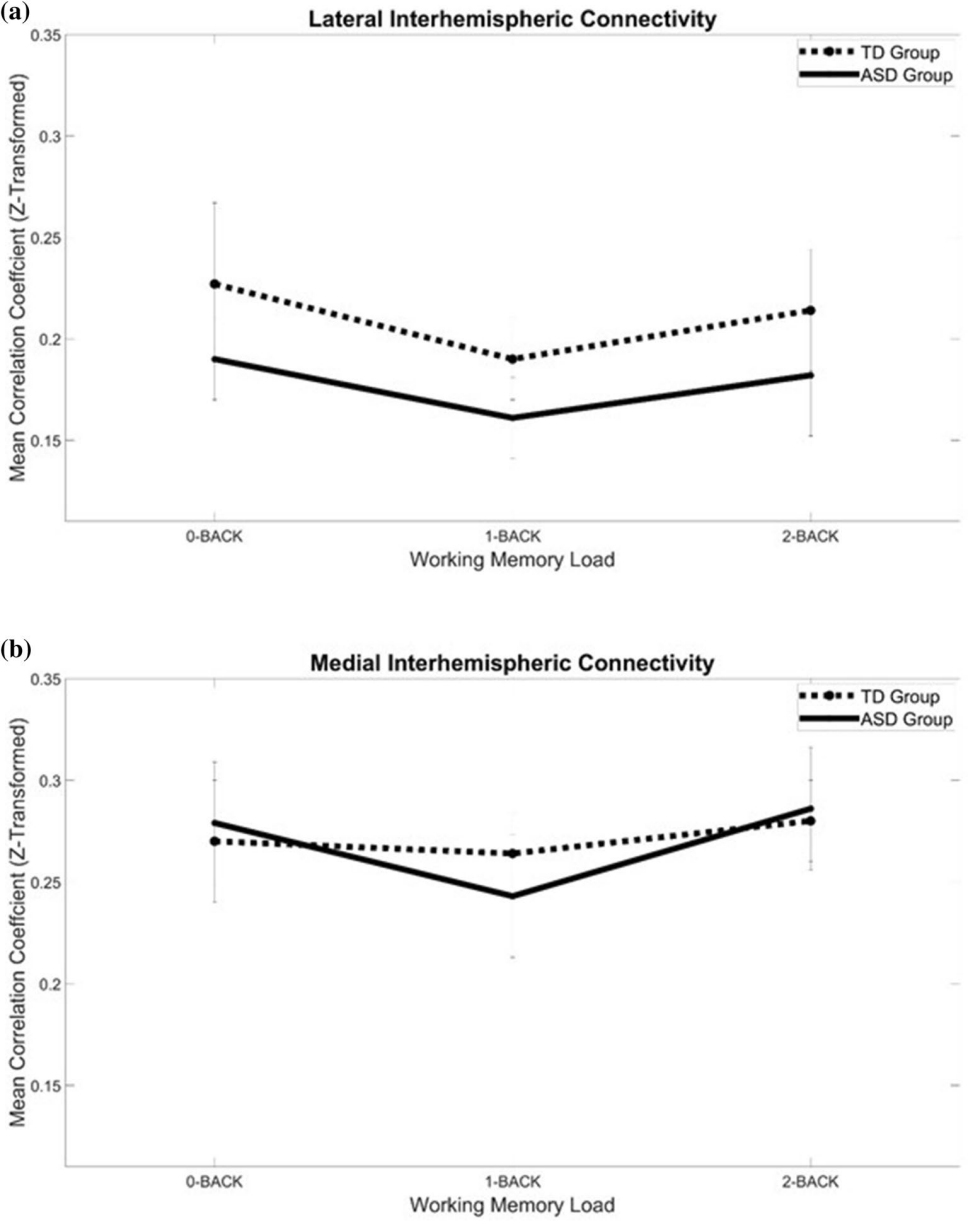
The effect of n-back task load on the (**a**) lateral and (**b**) medial interhemispheric functional connectivity changes in the prefrontal cortex in TD and ASD.

Regarding interhemispheric connectivity (Fig. 5), a 3 (load) × 2 (region) × 2 (group) mixed ANOVA showed that only the main effects of load (*F*_2,88_ = 3.22, *p* = .045, $\eta_{p}^{2} =$0.07) and region (*F*_1,44_ = 30.26, *p* < .001, $\eta_{p}^{2} =$0.41) were significant. None of the effects involving group was significant (*p*s > .27).

### Effects of n-back load on prefrontal activation

To complement the functional connectivity analysis, mean changes in HbO (i.e., activation) in the PFC during the *n*-back task were examined (Table 5). A 3 (load) × 2 (hemisphere) × 2 (region) × 2 (group) mixed ANOVA showed a significant main effect of group (*F*_1,44_ = 6.879, *p* = .012, $\eta_{p}^{2} =$0.14), with the ASD group showing higher activation than the TD group during the n-back task. Post hoc t-tests showed that higher activation in the ASD group was statistically significant only in the right medial region during the 0-back condition (*p* = .012) and in the right lateral region during the 1-back condition (*p* = .003); there were no differences in any ROI in the 2-back condition (ps > .039). Analysis with all TD (n = 24) and drug-free ASD (n = 14) individuals showed a similar trend of results (hemisphere x load x region x group 4-way interaction: F_2,72_ = 4.30, *p* = .045).

**Table 5:** Mean changes in oxyhaemoglobin concentration (Beta values) during the n-back task in TD (n = 24) and ASD (n = 22) individuals.

	Left Hemisphere		Right Hemisphere	
	TD	ASD	TD	ASD
	*M* (*SD*)	*M* (*SD*)	*M* (*SD*)	*M* (*SD*)
**0-back**				
Medial	1.184 (21.35)	9.608 (23.76)	− 8.035 (13.90)	8.300 (26.78)
Lateral	0.076 (15.55)	8.313 (40.26)	− 1.418 (23.07)	8.568 (27.02)
**1-back**				
Medial	− 1.972 (21.78)	4.931 (21.95)	− 6.214 (18.62)	− 1.714 (32.96)
Lateral	3.150 (20.88)	− 1.377 (33.97)	− 6.658 (17.12)	12.037 (22.88)
**2-back**				
Medial	1.938 (15.74)	12.644 (18.29)	− 2.661 (19.12)	9.921 (31.11)
Lateral	− 1.578 (19.17)	4.594 (27.09)	5.683 (20.95)	5.638 (31.14)

### Association between prefrontal functional connectivity and ASD symptomatology

Given that a significant interaction effect with group and WM load was identified in the right PFC, the functional connectivity values in the right medial and lateral PFC during the WM task were correlated with the SRS-2 total score. A significant positive correlation (rho = 0.460, *p* = .031) between the SRS-2 total score and right lateral PFC functional connectivity during the 0-back condition was detected in the ASD group only; other correlations remained nonsignificant (ps > .05).

## Discussion

The present study used fNIRS to examine the effects of WM load on PFC connectivity during the number *n*-back task in children aged 8–12 with high-functioning ASD. Compared to the age-, IQ-, sex- and handedness-matched TD children, the children with ASD exhibited slower performance during the “low load” (i.e., 0-back) condition only. Additionally, there were differential load-dependent functional connectivity changes in the lateral and medial PFC specifically in the right hemisphere but not in the left hemisphere. Our results suggest that individuals with ASD exhibit WM deficits, which is specifically mediated by the reduced information processing efficiency underpinned by abnormal functional connectivity in the right prefrontal cortex.

From the behavioral data, our finding of overall increased RTs during the n-back task among the children with ASD was consistent with previous observations of slower processing speed across various cognitive tasks in ASD^16,62–64^. It is well known that RT is a measure of the speed of information processing^65,66^, which represents the maximum rate at which a cognitive operation can be executed^67^. In the present study, the children with ASD needed more time to achieve a similar performance level as the TD children, a pattern that corroborates a large-scale *n*-back study with adults with ASD^68^. Despite showing a significant between-group difference with ANOVA, we are aware of the nonsignificant between-group RT differences (after Bonferroni corrections) for medium and high WM load conditions. The lack of a significant between-group difference might be attributed to the limited sample size and inherent heterogeneity within the ASD population regarding WM functioning^60^, which is subject to further research. The difference in reaction time between the ASD and TD groups is consistent with previous findings when ASD perform other executive functioning tasks (e.g., verbal fluency task). For instance, revealed that impaired verbal fluency performance is evident only when there is limited time given to ASD individuals; when they are given adequate time for task completion^69^, they could perform equally well when compared to their TD counterparts. These findings imply that the impairment of WM in ASD is mediated by abnormal information processing in the brain, providing evidence alongside a number of previous studies (e.g.,^70,71^). However, the accuracy rates across different WM loads were not significantly different between the two groups, which was consistent with previous studies involving high-functioning autism^33,72^. This could possibly be explained by the phenomenon that high-functioning ASD individuals tend to achieve complex WM task completion by utilizing their general intellectual capacities as compensation^20,73^.

Regarding fNIRS data, the children with ASD, compared to TD controls, exhibited aberrant load-dependent intrahemispheric functional connectivity and activation patterns within the right PFC. Specifically, when comparing the 0- and 1-back loads, visual inspection of data showed an increasing trend in functional connectivity in the right medial PFC in the TD group, while the ASD group showed a a decreasing trend of right medial intrahemispheric connectivity on load (Fig. 4d). The right medial PFC has been documented to be the key hub for WM maintenance^74^, learning and recalling learned associations between context, events and corresponding adaptive responses^75^. Additionally, the right hemisphere has been heavily implicated in vigilance or sustained attention^76^. Thus, disrupted connectivity within the right medial PFC may interfere with these cognitive processes and underlie the overall slowing in information processing in ASD. In fact, our finding of right-lateralized abnormalities during the WM task is consistent with previous studies showing abnormal lateralization patterns in ASD^60^. More importantly, our results have shown a positive correlation between functional connectivity in the right lateral PFC during the 0-back task and the SRS-2 total score, indicating that more impaired overall social functioning is associated with greater right lateral PFC functional connectivity during the low WM-load condition. Given that the low-load condition in the n-back task corresponds to a two-alternative forced choice task tapping simple visual information processing^77^ and that a more connected neural network reflects more effortful information processing^78^, our results may be interpreted as a more effortful processing of incoming information for individuals with more impaired social functioning, which is consistent with the theory attributing ASD behavioral manifestation to abnormal information processing^14^. However, it should be noted that we did not find a significant difference in laterality index between the ASD and the TD groups. This could be due to the fact that laterality indexes varied greatly among ASD individuals, showing both high and low scores^60^, as reflected by the large standard deviation of our laterality index data. Further studies are warranted to aid our understanding of the abnormal laterality pattern during WM tasks. Interestingly, our results show that trending functional connectivity differences occur in the 1-back load for the right medial PFC, while a significant reaction time difference (after Bonferroni correction) occurs in the 0-back load. A possible reason to explain this discrepancy is that the 1-back condition might also be challenging for TD children, and a previous large-scale normative study has shown that n-back performance varies greatly in TD children aged between 8 and 12^79^, the age group of our current sample. The inherently large heterogeneity in the 1-back condition may contribute to a less significant difference between TD and ASD.

This study is one of the first to have applied fNIRS to clarify neural processing during WM processing in ASD. Consistent with previous fMRI studies with adults with ASD^32,33^, one recent study by Yeung et al.^46^ showed that adolescents with ASD exhibited increased right lateralization of PFC activation, which was positively associated with WM abilities, in response to increasing WM load (2-back > 0-back) during an *n*-back task similar to the one employed in the present study. Specifically, the present study found significant right lateral PFC hyperactivation in the 1-back condition. Given the role of the lateral PFC in error monitoring, which is the process of checking task performance over time for quality control and adjusting behavior^80^, our results could imply an overall increase in effort for error monitoring in ASD during the *n*-back task. Interestingly, we did not observe between-group differences in the 2-back condition, which was in contrast to previous studies. The discrepancy in findings is likely due to age differences. Given the large heterogeneity of n-back task behavioral performance in children aged 8–12^79^, it is reasonable to expect that the frontal activation pattern also changes across age, although how age modulates the frontal activation pattern during the n-back task remains poorly understood. Taken together, the evidence suggests the potential importance of considering the developmental context when studying WM processing in ASD. To our knowledge, this study is the first to apply fNIRS to study cortical connectivity and report altered PFC connectivity during WM processing in ASD. Because fNIRS possesses adequate temporal and spatial resolution and can be readily used in a natural setting, it is a promising tool to study cortical connectivity in ASD.

While the findings in the present study have shed new light on the association between WM load and neurophysiological activities in children with ASD, the following limitations should be noted. First, it focused only on the PFC. It is well known that WM is supported by distributed frontal-parietal networks^21^. Thus, future work would benefit from examining the effect of WM load on functional connectivity between frontal and parietal regions. Second, we recruited only children with ASD. While a restricted age range had the benefit of increasing the homogeneity of the study sample, age may be an important factor that affects the behavioral and neurophysiological results, given the nonlinear differences in brain development between TD and ASD individuals across the lifespan^81,82^. Finally, we recruited only boys with ASD. Future studies are needed to determine whether the present findings are generalizable to females with ASD.

## Conclusion

The present study reported WM deficits and altered PFC connectivity during an *n*-back task in children with ASD. Because WM load influenced the pattern of PFC connectivity within the right but not the left PFC, this study highlights the importance of considering WM load to clarify the neural mechanisms underlying WM processing in ASD. Taken together, the literature implicates the right PFC in WM functioning in ASD^32,46^. There is some preliminary evidence that transcranial direct current stimulation^83^ and transcranial magnetic stimulation^84^ improve clinical and cognitive symptoms in individuals with ASD. Given that WM supports a variety of complex cognitive functions^2^, future research exploring the effectiveness of neurostimulation over the right PFC to mitigate WM problems in ASD is warranted.
